# Intestinal parasitosis in relation to CD4 count and anemia among ART initiated patients in St. Mary Aksum general hospital, Tigray, Ethiopia

**DOI:** 10.1186/s12879-019-3989-0

**Published:** 2019-04-27

**Authors:** Tuom Gebrewahid, Gebretsadkan Gebrekirstos, Mebrahtu Teweldemedhin, Hailay Gebreyesus, Abrham Awala, Kiros Tadla

**Affiliations:** 1 Department of Medical Laboratory Sciences, College of Health Sciences, Aksum University, P.O. Box 298, Aksum, Tigray Ethiopia; 2 Department of Public Health, College of Health Sciences, Aksum University, Aksum, Tigray Ethiopia; 3 Department of Medical Parasitology and Entomology, Institute of Biomedical, College of Health Sciences, Mekelle University, Mekelle, Tigray Ethiopia

**Keywords:** Intestinal parasites, CD4 counts, HIV/AIDS, Anemia, ART, Ethiopia

## Abstract

**Background:**

The geographical distribution of intestinal parasites with conditions of poverty in most countries of sub-Saharan Africa coincides with that of HIV/AIDS. However, there is paucity of studies investigating the relationship between intestinal parasitic infections with CD4 counts and anemia in HIV/AIDS patients starting Antiretroviral Therapy (ART) in this region particularly and in Ethiopia in general. The aim of this study was to determine the prevalence of intestinal parasitic infections in relation to CD4 count and anemia among ART-initiated patients in St. Mary Aksum General Hospital, Tigray, Ethiopia.

**Methods:**

A cross-sectional study was conducted among randomly selected 242 ART-initiated participants during February to April 2017 in St. Mary Aksum General hospital. Data was collected using structured questionnaire and laboratory examination. Logistic regression was applied to assess any association between explanatory factors and outcome variables (*P* values < 0.05).

**Result:**

The overall prevalence of intestinal parasites was 26.4% and among the six types of parasitic genera identified *Entamoeba histolytica/dispar* (18.6%) and *Giardia lamblia* (2.1*%*) were the leading. According to the multivariate analysis, lack of hand washing before meal, eating uncooked vegetables, history of taking anti-parasite medication, stool consistency, and anemia were strongly associated with intestinal parasitosis.

**Conclusion:**

There was a high prevalence of intestinal parasites among HIV positive individuals. Intervention measures such as deworming, improving hygiene and sanitation practices should be strengthened to reduce the burden of intestinal parasites among people living with HIV.

**Electronic supplementary material:**

The online version of this article (10.1186/s12879-019-3989-0) contains supplementary material, which is available to authorized users.

## Background

Worldwide, there are an estimated 3.5 billion people infected with intestinal parasites and out of them around 450 million were suffering from its illness [1]. In Sub-Saharan Africa, Intestinal parasitic infections are among the major public health problems causing morbidity and mortality [2]. In most countries of the sub-Saharan Africa, the burden of intestinal parasites are increasing with the immergence of HIV/AIDS [3] because the CD4 depletion and hematological abnormality secondary to HIV/AIDS predisposes opportunistic infections associated with intestinal parasites [4].

The clinical spectrum of the disease caused by the intestinal parasites particularly among Human Immunodeficiency Virus (HIV) positive individuals can be asymptomatic to severe infection [5]. Infection can cause decreased appetite, intestinal blockage and malabsorption, vitamin A deficiency, anemia, impaired growth and cognitive development [6, 7]. Moreover, chronic immune activation secondary to intestinal parasitic infections could increase host susceptibility, thereby promoting HIV/AIDS disease progression [8, 9].

Anemia is the commonest hematological abnormality in patients with HIV. It is associated with increased morbidity and is an indicator of poor prognosis among people with advanced HIV disease [10, 11]. Parasitic infections are commonly associated with anemia due to generally poor standard of hygienic practices in tropical Africa [12]. Studies indicated that around 43% of patients enrolled on antiretroviral therapy were having lower hemoglobin (anemia) at baseline; it is found to be the strongest predictors of mortality among these patients [13].

Intestinal parasites are common in Ethiopia because of the poor hygiene and sanitation practices [14]. It is reported that outpatient morbidity is majorly caused by parasitic infections in Ethiopia [15]. Investigation of the magnitude of intestinal parasites and identification of their potential risk factors is critically important for the management and control of both HIV and intestinal parasitic diseases. Since specific vaccine for the control of this parasite infections is not yet implemented, focusing on other prevention measures such as avoiding exposure to the parasite, and maintaining immune competence of the subject has paramount importance [16]. Most of the parasites are transmitted by fecal-oral route, through direct contact with infected persons, or by ingestion of contaminated food or water containing eggs and cysts of the parasite [17]. It is known that people living with HIV/AIDS are at higher risk of parasitic infections due to the decreased immunity. This is believed to decrease after immune reconstitution due to ART treatment [18]. Although, anemia and intestinal parasitic infections have been reported as co-morbidities in HIV infected patients [19], there is paucity of information in Ethiopia of the co-existence of these triple burdens and their association with CD4+ T cell levels among HIV infected patients in the country particularly in Tigray.

## Methods

### Study area, period and design

A cross sectional study was conducted in St. Mary Aksum General hospital from February to April 2017. The hospital is found in Aksum town located 1032 Kilometers north of Addis Ababa the capital of Ethiopia. The town is situated in central zone of Tigray with latitude and longitude of N 14° 7′ 47.00“ E 38° 42’ 57.00” and the elevation of the town is 2132 m above sea level. The average annual rainfall of the area is 729.7 mm and temperature of 18.40 °C [7]. The hospital serves for an estimated population of 1.2 million and provides many health care services including counseling, HIV testing and ART.

### Population

All HIV positive patients who enrolled to St. Mary Aksum General hospital’s ART clinic were considered as the source population. All HIV patients on ART enrolled and who came to ART clinic for their follow up during the study period was the study population.

### Sample size determination and sampling techniques

The sample size was determined using the single proportion formula:

$$ {\displaystyle \begin{array}{l}\mathrm{n}\kern0.5em =\kern0.5em \frac{{\mathrm{Z}}^2\kern0.5em \mathrm{P}\kern0.5em \left(1\kern0.5em \hbox{-} \kern0.5em \mathrm{P}\right)}{{\mathrm{d}}^2}\\ {}\mathrm{Where}:\kern0.5em \mathrm{n}\kern0.5em =\kern0.5em \mathrm{sample}\ \mathrm{size}\kern11em \mathrm{Z}=\mathrm{Z}\ \mathrm{statistic}\ \mathrm{for}\ \mathrm{a}\ \mathrm{level}\ \mathrm{of}\ \mathrm{confidence}\\ {}\kern6em \mathrm{d}=\mathrm{precision}\kern12.5em \mathrm{P}\kern0.5em =\kern0.5em \mathrm{expected}\ \mathrm{prevalence}\ \mathrm{or}\ \mathrm{proportion}\end{array}} $$

According to a research done in Dessie Hospital, the prevalence of intestinal parasites in-ART patients was (17.6% [20]. A 95% level of confidence (z = 1.96) and 5% margin of error (d) was used. With these assumptions the minimum sample size required for the study was 223 and with 10% added for non-respondents, the required sample size was increased to 245 and finally 242 study participants were recruited during the study period.

The study participants was selected by simple random sampling technique from the daily follow up list of HIV patients (15–30 patients) that come in a day to the ART clinic. The 242 participants were tested in two months with 6 individuals in a day selected by lottery method.

### Variables

**Dependent Variables**: Prevalence of intestinal parasite

### Independent variables

Socio-demographic factors such as age, sex, religion, address, educational status, marital status and Clinical Characteristics such as CD4 count, anemia time of ART start, World Health Organization (WHO) disease stage, Type of Antiretroviral (ARV) drug, stool consistency, diarrhea, environmental and personal hygiene such as habit of hand washing before meal, habit of eating uncooked vegetables, source of drinking water, existence of toilet, habit of hand washing after toilet, participation on agricultural practices were the independent variables.

### Inclusion criteria

Individuals (all age groups) who were on ART and volunteered to participate were included in the study. On the other hand, those who were taking anti-parasitic medications within the past two weeks except Cotrimoxazole and those with history or diagnosis of any other acute or chronic disease causing immunosuppression and/or anemia such as autoimmune hemolytic anemia, Sickle Cell Anemia, Aplastic Anemia and Thalassemia were excluded from the study. Additionally, individuals who were under ART regimen containing Zidovudine were excluded from the study because it is known to produce anemia.

### Data collection procedure

Consent was taken from all of the participants above the age of 18 years. For the participants who were blow the age 18 years, assent was taken from their parents or guardians. A structured questioner was used to assess the Socio-demographic and clinical characteristics as well as Environmental and personal hygienic related variables. Trained Bachelor of Science Nurses collected the data by direct interview of the participants and by reviewing their medical record. On the other hand, trained Medical laboratory professionals performed the complete blood count and the CD4+ T cell count using fully automated hematology analyzer (Cell-Dyn 1800) and FACScalibur respectively.. Adequate stool specimen was collected from each participant and examined macroscopically for the presence of any adult intestinal parasites, for consistency and for any other physical abnormalities. Wet mounts were prepared and examined under light microscope at 10X and 40X objectives. Furthermore, Formol–Ether concentration and Modified Ziehl-Neelsen staining was performed as per the standard operating procedures (see Additional file 1). The remaining portion of the stool specimen was preserved in 10% formalin for repeating the tests whenever required and for further analysis [21, 22].

### Quality control

All laboratory analyses were carried out using standard operating procedures. Site assessment and pre-test was done prior to the actual data collection and adjustment was made accordingly. Adequate stool specimen (40 g of formed stool and 10 ml of diarrheic stool) was collected using carefully labeled, dry, leak proof and grease free transparent stool caps. The specimen was kept free from water, soil, and urine contamination. Specimens contaminated with water, urine and soil was rejected and the study participants were requested to bring another. Positive and negative controls were used to check the quality of the microscope and the staining solutions. Direct stool examination was performed within 30 min to avoid delay. All microscopic findings and questionnaire based information were encoded and reported appropriately. CD4+ T cell categorization and anemia definition was made based on the WHO criteria.

### Data analysis and interpretation

For data entry and analysis SPSS version 20.0 statistical software was used. Overall socio demographic, clinical characteristics and specific prevalence was calculated using descriptive statistics of the sample through frequencies and cross tabulations. The association or crude odds ratio of parasitic infections with the independent variables was calculated using bivariate logistic regression analysis. Association was established by multivariable logistic regression analysis; the 95% confidence intervals (CI) and *P* < 0.05 was considered for statistical significance.

## Result

### Socio-demographic characteristics of the participants

From the total of 245 study participants, 242 (98.8%) were able to provide stool specimens. Analysis was based on the 242 subjects and of these, 147(60.7%) were females. The age of the participants ranged from 8 to 66 years with a mean age of 38 ± SD (37.58 ± 10.9) (Table 1).

**Table 1:** Socio demographic characteristics and prevalence of parasitic infection in ART initiated HIV patients in St. Mary Aksum General Hospital, Tigray, Ethiopia, 2017

Variable		Frequency	Percentage %	Positive to IPIs, n (%)
Age in year	< 15	12	5.0	4(33.3)
	15–29	26	10.7	11(42.3)
	30–39	95	39.3	24(25.3)
	40–49	74	30.6	18(24.3)
	50+	35	14.5	7(20.0)
Gender	Male	95	39.3	28(29.5)
	Female	147	60.7	36(24.5)
Religion	Orthodox	214	88.4	58(27.1)
	Muslim	28	11.6	6(21.4)
Residence	Urban	222	91.7	54(24.3)
	Rural	20	8.3	10(50.0)
Marital status	Single	27	11.2	10(37.0)
	Married	110	45.5	35(31.8)
	Divorced	89	36.8	17(19.1)
	Widow	16	6.6	2(12.5)
Educational status	Illiterate	64	26.4	14(21.9)
	Elementary	100	41.3	31(31.0)
	Secondary	61	25.2	18(29.5)
	College And University	17	7.0	1(5.9)
Occupation	Civil servant	36	14.9	8(22.2)
	Merchant	47	19.4	11(23.4)
	Farmer	15	6.2	7(46.7)
	Daily laborer	84	34.7	23(27.4)
	Student	18	7.4	6(33.3)
	House wife	42	17.4	9(21.4)
Total		242	100	64(26.4)

Not: *IPs* Intestinal Parasites

### Prevalence of intestinal parasites among ART patients

**Fig. 1:**
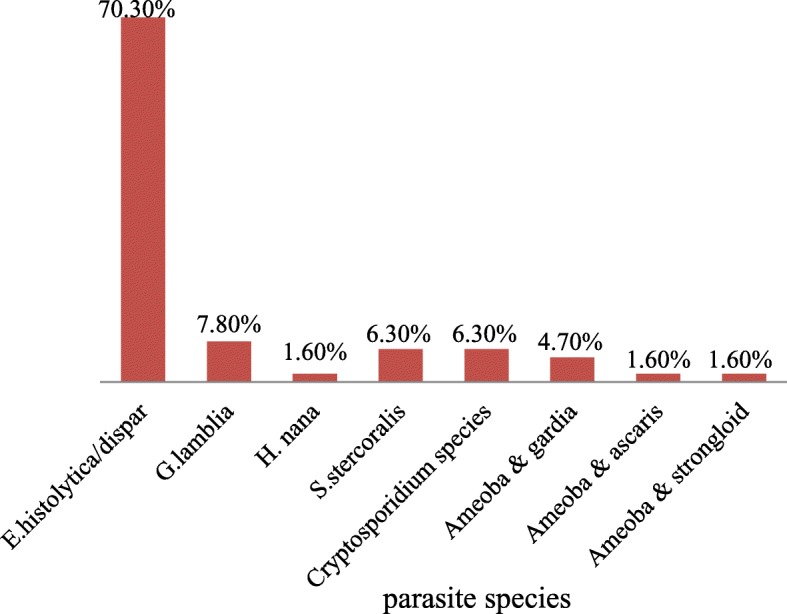
Prevalence of different species of intestinal parasites in the study participants in St Mary Axum General hospital, 2017

Of the 242 study participants one or more intestinal parasites (IPs) were detected in 64 (26.4%) individuals. Of the 64 infected patients, 92.2% (59/64) harbored single parasite and 7.8% (5/64) harbored more than one parasite. Six types of parasitic genera were identified from the study participants (Fig. 1). The highest prevalent intestinal parasite was *E. histolytica/dispar.*Among the opportunistic parasites, only *Cryptosporidium species* were detected in participants whose CD4 counts were less than 500 cells /μL.

### Prevalence of intestinal parasites in relation to the different risk factors

Out of the 242 participants, 191 (78.9%) of them practiced hand washing before meal, 183(75.6%) ate cooked vegetables. From the participants who did not have a habit of hand washing by soap before meal, 52.9%(27/51) were positive for parasitic infection (Table 2).

**Table 2:** Prevalence of intestinal parasites in relation to the different risk factors in St. Mary Aksum General Hospital, from March to April 2017, Tigray, Ethiopia

Variables		Frequency	Percentage (%)	Positive to IPIs, n (%)
Practice of hand washing before meal	Yes	191	78.9	37(19.4)
	No	51	21.1	27(52.9)
Practice of eating uncooked vegetables	Yes	59	24.4	26(44.1)
	No	183	75.6	38(20.8)
Practice of eating raw meat	Yes	14	5.8	5(35.7)
	No	128	94.2	59(25.9)
Water source of drinking	Tap water	232	95.9	62(26.7)
	River	10	4.1	2(20.0)
Presence of toilet	Yes	237	97.9	62(26.2)
	No	5	2.1	2(40.0)
Practice of hand	Yes	240	99.2	63(26.2)
washing after toilet	No	2	.8	1(50.0)
Practice of finger nails trim	Yes	213	88.0	59(27.7)
	No	29	12.0	5(17.2)
Participate in agriculture	Yes	39	16.1	15(38.5)
	No	203	83.9	49(24.1)
History of medication for intestinal parasite	Yes	146	60.3	30(20.5)
	No	96	39.7	34(35.4)
Total		242	100	64 (26.4%)

Note: *IPs* Intestinal parasites

### Clinical characteristics and laboratory profile of ART patients

Among all study participants, 231(95.5%) started ART before one year and 236(97.5%) were WHO HIV stages I(Table 3). The majority of the participants (55.4%) had CD4 counts over 500 cells/μl CD4Of those having CD4 count < 200cells/μl, 46.2% were positive for intestinal parasites. The overall proportion of subjects with diarrhea was 11.6% (28/242) and parasitic infection among patients with diarrhea was higher 46.4% (13/28) than patients without diarrhea 23.8% (51/214) (*p* = 0.013). From the total of 242 ART initiated HIV patients, 27(11.2%) were anemic and 15/27(55.6%) had IPs. Among subjects with anemia, 20/27(74.1%) had mild anemia (hemoglobin level 11.0–11.9 g/dl, 7/27(25.9%) were moderately anemic (hemoglobin level 8.0–10.9 g/dl) and no participant had severe anemia (Table 3).

**Table 3:** Clinical Characteristics and laboratory profile of ART patients in relation to the parasite positivity rate in St. Mary Aksum General Hospital, from March to April 2017 Tigray, Ethiopia

Variables		Frequency (%)	Positive to IPIs, n (%)
Start of ART	Less than one year	11(4.5)	4(36.4)
	before one year	231(95.5)	60(26.0)
Current WHO HIV stage	I	236 (97.5)	63(26.7)
	II	3(1.2)	1(33.3)
	III	3(1.2)	0(0.0)
Type of ARV drug	First line	224(92.6)	57(25.4)
	Second line	18(7.4)	7(38.9)
Category of CD4 now	< 200	39(16.1)	18(46.2)
	200–500	69(28.5)	16(23.2)
	500+	134(55.4)	30(22.4)
Stool consistency	Formed	69(28.5)	8(11.6)
	Soft	101(41.7)	26(25.7)
	Loose	44(18.2)	17(38.6)
	Diarrheic	28(11.6)	13(46.4)
Diarrhea condition	No diarrhea	214(88.4)	51(23.8)
	Chronic	17(7.0)	8(47.1)
	Acute	11(4.6)	5(45.5)
Anemia status	No anemia	215(88.8)	49(22.8)
	Mild	20(8.3)	10(50.0)
	Moderate	7(2.9)	5(71.4)
Presence of Parasites	Yes	64(26.4)	64(26.4)
	No	178(73.6)	178(73.6)
Total		242(100)	64 (26.4%)

Note: *ART* Antiretroviral Therapy, *ARV* Antiretroviral, *IPs* Intestinal parasites

### The factors associated with intestinal parasite among ART initiated HIV/AIDS patients attending ART clinic

Socio demographic and environmental variables in relation to IP prevalence were analyzed by using binary logistic regression model. In the bivariate analysis, age, gender, occupation, religion, marital and educational status of the participants did not show significant association with prevalence of intestinal parasites. However, residence, habit of washing before meal, practice of eating uncooked vegetable, taking anti-parasite medication, CD4 category, anemia, anemia status, stool consistency, diarrhea and diarrhea condition showed significant association with prevalence of intestinal parasites. After adjusting for all socio-demographic characteristics and potential confounding variables using multivariate logistic regression, intestinal parasitosis was significantly associated with d not washing hands before meal [AOR = 4.668; 95% CI: 2.306–9.446], eating uncooked vegetable [AOR = 2.756; 95% CI: 1.377–5.514], history of taking anti-parasite medication [AOR = 2.053; 95% CI: 1.083–3.895], stool consistency, diarrhea condition (AOR = 4.119; 95% CI: 1.237–13.708) and anemia [AOR = 2.739; 95% CI: 1.023–7.334] (Table 4)..

**Table 4:** Prevalence of intestinal parasites and its association with socio-demographic, clinical and other risk factors among ART initiated HIV patients (*n* = 242) in St. Mary Aksum General Hospital, Tigray, Ethiopia in 2017

Characteristics		No. of study participant	No. of individuals with IPIs (%)	COR (95% CI), *p*-value	AOR (95%CI), p-value
Residence	Urban	222	54(24.3)	1	1
	Rural	20	10(50.0)	.311(1.229–7.874), .017	1.597(.516–4.941), .417
Wash hands before meal	Yes	191	37(19.4)	1	1
	No	51	27(52.9)	4.682(2.428–9.029), .000	4.668(2.098–8.933), **< 0.01**
Eat uncooked Vegetable	Yes	59	26(44.1)	3.006(1.608–5.622), .001	2.756(1.377–5.514), **.005**
	No	183	38(20.8)	1	1
Anti-parasite Medication	Yes	146	30(20.6)	1	1
	No	96	34(35.4)	2.120(1.188–3.786) .011	2.043(1.067–3.895), **.028**
CD4 category	< 200	39	18(46.2)	2.971(1.405,6.286), .004	.512(.134–1.955), .328
	200–500	69	16(23.2)	1.047(.524–2.089), .897	.518(.221–1.215), .131
	500+	134	30(22.4)	1	1
Anemia	Yes	27	15(55.6)	4.235(1.859–9.646), .001	2.739(1.023–7.334), **.045**
	No	215	49(22.8)	1	1
Stool consistency	Formed	69	8(11.6)	1	1
	Soft	101	26(25.7)	2.643(1.117–6.256), .027	2.374(.951–5.927), .064
	Loose	44	17(38.6)	4.801(1.848–12.472), .001	2.652(.921–7.637), .071
	Diarrhea	28	13(46.4)	6.608(2.321–18.815), .000	4.119(1.237–13.708), **.021**
Diarrhea condition	No diarrhea	214	51(23.8)	1	1
	Chronic diarrhea	17	8(47.1)	2.841(1.041–7.745), .041	1.234(.204–7.473), .813
	Acute diarrhea	11	5(45.5)	2.663(.780–9.092), .118	

Note: *AOR* Adjusted Odds Ratio, *COR* Crude Odds Ratio; 1: referent

Variables with a *p* value in boldface are statisticaly significant

## Discussions

This cross-sectional study aimed to assess the magnitude of parasitic infection among ART patients and to identify the associated factors. The overall prevalence rate of intestinal parasites among ART initiated HIV/AIDS patients was 26.4%. This is almost similar to the prevalence of IPs among patients on ART in Bahir-Dar 25.5%, Addis Ababa 27.14%, Butajira 26.6% and Hiwot Fana, Eastern Ethiopia 28.6% [1, 23–26].

This finding is higher than the prevalence of intestinal parasites reported from Desie, Ethiopia 17.6% and Nigeria 5.3, 8.2% [20, 25, 27–29]. This difference could be due to the difference in diarrheic status, immune status, environmental hygiene and personal hygiene of the study participants. However, it is lower than those from Adigrat 56%, Jimma 39.56%, Fiche Hospital 55.1%, Dire Dawa and Afar 48% of Ethiopia and Cameroon 59.52, 82.6% [30–36]. This low prevalence of parasite in this study might be due to the difference in sample size, the geographic difference, and study period in which nowadays there is a better awareness of the patients about intestinal parasite infection and their cause. It might be also due to improved care provided to people living with HIV/AIDS and adherence to ART. The frequent advice given by healthcare providers for HIV positive patients during their frequent visit to ART clinic could contribute for lowering magnitude of intestinal parasitic infection.

In this study, the prevalence of *E. histolytica/dispar* and *G. lamblia* was significantly higher than the helminthes which is also consistent with other previous studies conducted in Ethiopia (Bahir Dar, Desie, Hawassa) and Cameroon [1, 20, 32, 33]. The overall prevalence of *E. histolytica/dispar* in this study was found to be 18.6% (45/242). This is consistent with previous results (19.3%) in Felegehiwot Referral Hospital and (20%) Bahir Dar, Ethiopia [24, 37]. The higher existence of protozoan may be that the participants did not take antiprotozoan dugs in their follow up.

IPs and individual hygiene and sanitation practices were very clearly related. The habit of not washing hands with soap before meal and the practice of eating uncooked vegetable contributed to the higher proportion of IPIs. This study showed that ART patients who did not have the habit of washing before meal were almost 4.7 times more likely (AOR = 4.682, 95% CI = 2.428, 9.029) to have parasitic infection than those who had habit of hand washing before meal. Likewise, those with the habit of eating uncooked vegetables were 3 times more likely (AOR = 3.006, 95% CI = 1.608,5.622) to have intestinal parasitic infection than those who did not eat uncooked vegetable; this finding is in line with the study done in Adigrat Hospital and Addis Abeba [35, 38].

Subjects who had diarrhea were 4 times more likely (AOR = 4.119, 95% CI = 1.237, 13.708) to have had intestinal parasitic infection than those who had formed stool and result is Similar with studies done in Hawassa, Hiwot Fana Ethiopia and Cameroon [1, 33, 39]. This could be due to the diarrheic are more immunocompromised by the loss of electrolytes with diarrhea consequently infected with intestinal parasites could not eliminate these. Non medicated participants were 2 times more likely to had parasite than medicated and similar result were found in Brazil [40] this could due to medicated participants with anti-parasites could restore the immunity of the patient.

In agreement with other studies except for *Cryptosporidium species,* opportunistic coccidian parasitic infections were not detected in the present study [38]. ART have been documented to improve immune status of the patients, thus prevent the occurrence of opportunistic parasitic infections [35]. It has also been suggested that HAART helps in eradicating opportunistic protozoan infections and that it is associated with the increase of CD4+ cells [38].

The decreased rate of cryptosporidiosis (1.7%) and absence of other opportunistic protozoan infections among patients with a CD4 T cell count < 200 cells/μl may indicate the success of intervention efforts in reducing the significance of opportunistic intestinal parasitic infections in the specified high risk group. This was also supported by many similar studies conducted in different settings showing a lower rate of cryptosporidiosis among HIV-infected patients on ART [1, 20, 41]. Isospora belli and Cyclospora were not detected in the present study which could be due to the Co-trimoxazol prophylaxis which is supplied with the ART drugs [42] and low number of oocysts excreted Additionally, the improvement of immune status associated with the ART treatment might have decreased the occurrence of opportunistic protozoan infections [1, 24, 35, 36].

A high prevalence of intestinal protozoa than intestinal helminthes (56 (89.1%) vs. 5 (11.9%) was observed in this study. This was in line with studies conducted elsewhere [20, 33]. Most of the participants were given anti-helminthes for deworming purpose before months. This may be the reason for the low prevalence of helminthes. Another reason might be reduced egg excretion may cause low detection rate of these parasites in fecal sample resulting in lower magnitude of helminthes [23].

Intestinal parasitosis was more frequent in participants that had diarrhea, and diarrhea was more common in participants with CD4 counts of < 200 cells/μL. This is in agreement to the studies done in Hawassa, Hiwot Fana Ethiopia and Cameroon [1, 33, 39]. Anemia was more in participants who had CD4 less than 200 cells/ μL and intestinal parasitic infection were more in those participants. This was supported by another study in Nigeria [43] and this might be related to poor immunological recovery and high viral load.

In general, the reduction in the prevalence of parasitic infection in this study may be due to the current intervention measures on HIV-infected people. This may encourage public health officials and policy makers to further strengthen the existing HIV-related care and treatment programs to reduce morbidity and mortality associated with intestinal parasites. As a limitation of this study, Co-trimoxazole prophylaxis might have affected the detection of the parasite which responds to this antibiotic.

## Conclusion

There was high prevalence of intestinal parasites among HIV positive individuals, with most of the parasites found were protozoan parasites like *E. histolytica/dispar* and *G. lamblia.* However, the results in the current report were significantly lower when compared to other studies in the Country. The prevalence of IPIs was associated significantly with habit of washing hands before meal, eating uncooked vegetables, taking anti-parasite, anemia, CD4 count and stool consistency. The existing intervention measures should be strengthened and sustained in order to further reduce intestinal parasitic infections in people living with HIV/AIDS. Health education on improving personal hygiene and environmental sanitation should be given. Deworming and treatment of intestinal parasites of HIV/AIDS should be targeting protozoan parasites.

## Additional file

Additional file 1: Stool specimen standard operating procedures. (DOCX 14 kb)

## Abbreviations

AOR: Adjusted Odds Ratio
ART: Antiretroviral Therapy
ARV: Antiretroviral
CD4: Cluster of Differentiation 4
CI: Confidence Interval
COR: Crude Odds Ratio
HIV/AIDS: Human Immunodeficiency Virus/Acquired Immunodeficiency Syndrome
IPs: Intestinal Parasites
WHO: World Health Organization
