# Supplementary material for: Intestinal parasitosis in relation to CD4 count and anemia among ART initiated patients in St. Mary Aksum general hospital, Tigray, Ethiopia
Source: BMC Infect Dis. 2019 Apr 27;19:350. doi: 10.1186/s12879-019-3989-0 (PMC6486999; doi:10.1186/s12879-019-3989-0)
Supplement: Supplementary file 1 — Stool specimen standard operating procedures. (DOCX 14 kb) [file 12879_2019_3989_MOESM1_ESM.docx]

## S 1: Stool specimen standard operating procedures

**a) Direct wet mount**

**Principle:** Many parasites cause disease in man. Some of these parasites are excreted in stool; they are called intestinal parasites. Intestinal parasites can be identified by examination of fresh stool samples. In stool samples worms (eg. Ascaris lumbricoides) and segments of worms (e.g. Taenia species) visible to the eye can be find. By microscopic examination of fresh stool samples, we can find eggs (e.g. Hookworm) and larvae of worms (e.g. Strongyloides stercoralis). We also find protozoa trophozoites (e.g. Amoeba) and cysts (e.g. Cyclospora cayetanensis). In heavy and moderate infection, a direct smear examination with normal saline and/or iodine to stain cysts is usually sufficient. For light infections, a concentration of the stool sample might be required to find helminthes (worm) eggs and protozoa by microscopic examination.

**Procedure**

1. Place a drop of normal saline on a clean slide

2. Using a piece of stick, place a small amount of specimen, including blood and mucus in one end of the slide and cover it with a cover slide

3. First examine microscopically using 10 x objectives to give good contrast and use the 40x objective to identify trophozoites of protozoa.

**Reporting:** Report the name of the parasite found.

**b) Formalin-Ether concentration technique**

**Principle:** In the Ridley modified method, feces are emulsified in formol water, the suspension is strained to remove large fecal particles, ether or ethyl acetate is added, and the mixed suspension is centrifuged. Cysts, oocysts, eggs, and larvae are fixed and sedimented and the fecal debris is separated in a layer between the ether and the formol water. Fecal fat is dissolved in the ether.

**Procedure**

1. Emulsify 1g of stool in 4 ml of 10% Formol water in a tube using a stick

2. Add a further 3-4 ml of 10% formol water, cap the tube and mix well by shaking

3. Sieve the emulsified feces and collect the suspension in a beaker

4. Transfer the suspension to centrifuge tube and add 3-4 ml of diethyl ether or ethyl acetate

5. Mix the tube for 1 minute centrifuge at 3000 rpm for 1 minute

6. Discard the ether, fecal debris and formol water

7. Tap the bottom of the tube and mix the sediment

8. Add a drop of normal saline in a clean slide, add a piece of specimen and cover it with cover slide

9. First examine microscopically using 10 x objectives to give good contrast and use the 40x objective to identify cysts and ova of parasites and add iodine to the smear for staining([63](#_ENREF_63)).

**Formol- ether oocyst concentration technique**

Follow steps from 1 to 5 of the above method and continue as follows;

6. Centrifuge immediately at low speed, at 1000 rpm for 1 minute, remove the fluid from fecal debris and ether and transfer it to a centrifuge tube

7. Add formol water to make volume up to 10-15 ml and centrifuge at 3000 rpm for 5-10 minute

8. Remove the supernatant, tap the bottom of the tube, mix the sediment and examine using 40 x objectives.

**Principle:** This technique is useful for the identification of oocysts of the coccidian species (*Cryptosporidium*, *Cystoisospora*, and *Cyclospora*), which may be difficult to detect with routine stains such as trichrome. Unlike the routine Ziehl-Neelsen, this stain does not require the heating of reagents for staining.

**Procedure**

1. Prepare a smear from the sediment obtained by the formol ether oocyst concentration technique (see annex VI), air dry it, and fix the smear with methanol for 1 minuteand allow to dry

2. Stain with unheated carbol-fuschin for 30 minutes and wash off the stain with water

3. Decolorize with 1% acid alcohol for 1-2 minutes and wash off with water

4. Counter stain with 0.3% malachite green (or methylene blue) for 60 seconds and wash off with water

5. Air dry and examine under low power to detect oocyst and under oil immersion to identify them.

Interpretation: Small, round to oval, pink red stained bodies measuring 4–6 micrometer indicate oocysts.

**c) Modified Ziehl-Neelson (Z-N) staining method**

Principle: This technique is useful for the identification of oocysts of the coccidian species (*Cryptosporidium*, *Cystoisospora*, and *Cyclospora*), which may be difficult to detect with routine stains such as trichrome. Unlike the routine Ziehl-Neelsen, this stain does not require the heating of reagents for staining.

**Procedure**

1. Prepare a smear from the sediment obtained by the formol ether oocyst concentration technique (see annex VI), air dry it, and fix the smear with methanol for 1 minuteand allow to dry

2. Stain with unheated carbol fuschin for 30 minutes and wash off the stain with water

3. Decolorize with 1% acid alcohol for 1-2 minutes and wash off with water

4. Counterstain with 0.3% malachite green (or methylene blue) for 60 seconds and wash off with water

5. Air dry and examine under low power to detect oocyst and under oil immersion to identify them.

**Interpretation**: Small, round to oval, pink red stained bodies measuring 4–6 micrometer indicate oocysts.
